# Ubiquitination in macrophage plasticity: from inflammatory to immunometabolic pathways

**DOI:** 10.3389/fimmu.2025.1621328

**Published:** 2025-09-25

**Authors:** Jianing Liu, Magdalena Paolino

**Affiliations:** ^1^ Division of Neurogeriatrics, Department of Neurobiology, Care Sciences and Society, Karolinska Institutet, Stockholm, Sweden; ^2^ Department of Medicine Solna, Center for Molecular Medicine, Karolinska Institutet, Stockholm, Sweden; ^3^ Karolinska University Hospital, Stockholm, Sweden

**Keywords:** macrophage, polarization, ubiquitination, immunometabolism, inflammation

## Abstract

Macrophages are highly plastic innate immune cells whose polarization and effector functions are tightly linked to their metabolic programs. Ubiquitination, the post-translational modification that attaches ubiquitin chains to target proteins, plays a crucial role in regulating macrophage immunometabolism and phenotype transitions. In this mini-review, we summarize the current understanding of ubiquitin-dependent mechanisms that modulate macrophage polarization. We discuss how E3 ubiquitin ligases and deubiquitinases regulate key metabolic and signaling pathways, balancing pro-inflammatory and immunosuppressive states. Additionally, we describe the pathophysiological consequences of dysregulated ubiquitin-dependent control of macrophage polarization and its implications for disease. These insights underscore the importance of ubiquitination as a central modulator of macrophage function and its potential as a therapeutic target for controlling immunity in infections, inflammation, and cancer.

## 1 Introduction

**Figure 1:**
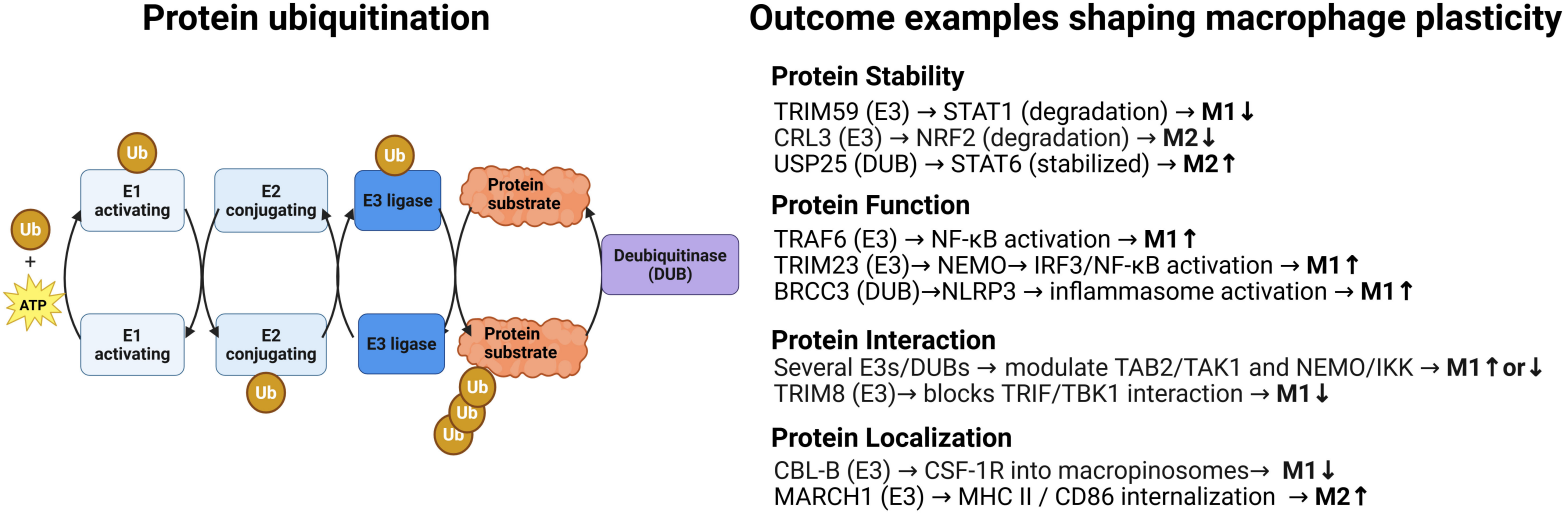
Protein ubiquitination: a dynamic and reversible posttranslational modification with multiples possible outcomes that modulate macrophage plasticity. Left. Schematic representation of the enzymatic steps involved in ubiquitination and deubiquitination. The ubiquitination process proceeds in three steps: activation of ubiquitin (Ub) by an E1 enzyme in an ATP-dependent manner, its transfer to an E2 conjugating enzyme, and its covalent attachment to a lysine residue on the substrate by an E3 ligase. Deubiquitinases (DUBs) reverse this process by cleaving isopeptide bonds either between ubiquitin and the substrate or within ubiquitin chains, thereby editing or removing ubiquitin modifications. For simplicity, only one type of ubiquitin chain is shown; in reality, substrates can be modified with mono-, multi-, or polyubiquitin chains, which may also be mixed or branched. This structural diversity enables a wide range of outcomes for the ubiquitinated substrate, including altered stability, degradation, trafficking, localization, activity, and protein–protein interactions. Right: Selected examples of substrate-specific outcomes, such as degradation, altered localization, modulation of activity, and changes in protein–protein interactions, illustrating how ubiquitin-mediated regulation contributes to macrophage plasticity. Additional ubiquitin enzymes, substrates, and regulatory outcomes are discussed in the main text and shown in **Figure 2**. Created in BioRender. Paolino, M. (2025) https://BioRender.com/i788nmb.

Precise control of protein function is essential for regulating cell signaling and fate. Ubiquitination, a versatile post-translational modification, achieves this by covalently attaching a 76-amino-acid ubiquitin protein to targets through an enzymatic cascade involving E1 activating enzymes, E2 conjugating enzymes, and E3 ligases (1) (**Figure 1**). Substrates are tagged with either single ubiquitin molecules or polyubiquitin chains of various linkage types, altering their stability, localization, or interactions (2, 3). Hundreds of E3 ligases confer substrate specificity, while deubiquitinating enzymes (DUBs) remove ubiquitin chains, creating a dynamic, finely tuned ubiquitin signaling system that regulates cellular processes.

Ubiquitination is essential for regulating immune cells, including innate immune cells. It is well established that ubiquitination controls key pro- and anti-inflammatory signaling pathways, such as NF-κB and interferon signaling (4), balancing homeostasis and activation to ensure robust yet restrained immune responses. More recently, it has become clear that ubiquitination integrates metabolic signals with inflammation regulation to fine-tune immune outcomes. This integration of immune phenotype with metabolic programming -referred to as immunometabolism- is a hallmark of macrophage biology. Their polarization into distinct tissue functions requires coordinated immune signaling and metabolic control (5–7). This principle is well illustrated by the two main polarized macrophage phenotypes: canonical pro-inflammatory M1-like macrophages, which rely on glycolysis, and anti-inflammatory M2-like macrophages, which depend on oxidative phosphorylation and fatty acid oxidation.

This mini-review highlights key studies illustrating how ubiquitin-dependent pathways orchestrate macrophage functional plasticity by not only regulating various inflammatory cascades, but controlling and integrating key metabolic circuits to shape functional outcomes, with significant implications for immune-mediated diseases.

## 2 Ubiquitination in macrophage functional diversity

Macrophage polarization refers to the ability of macrophages to adopt distinct functional programs in response to environmental signals, enabling them to coordinate diverse processes such as pathogen clearance, tissue repair, immune regulation, and inflammation resolution (8). Ubiquitination orchestrates signaling pathways that drive macrophage polarization across a spectrum of activation states, including pro-inflammatory M1-like and anti-inflammatory M2-like phenotypes, among others, as well as their functional plasticity—the ability to switch between these states. Various E3 ligases act as molecular on/off switches, promoting or inhibiting specific pathways, while deubiquitinases (DUBs) adjust responses by either sustaining signaling -by removing degradative ubiquitin marks- or terminating it -by removing activating ubiquitin chains (**Figure 1**).

### 2.1 Pro-inflammatory M1-like polarization

**Figure 2:**
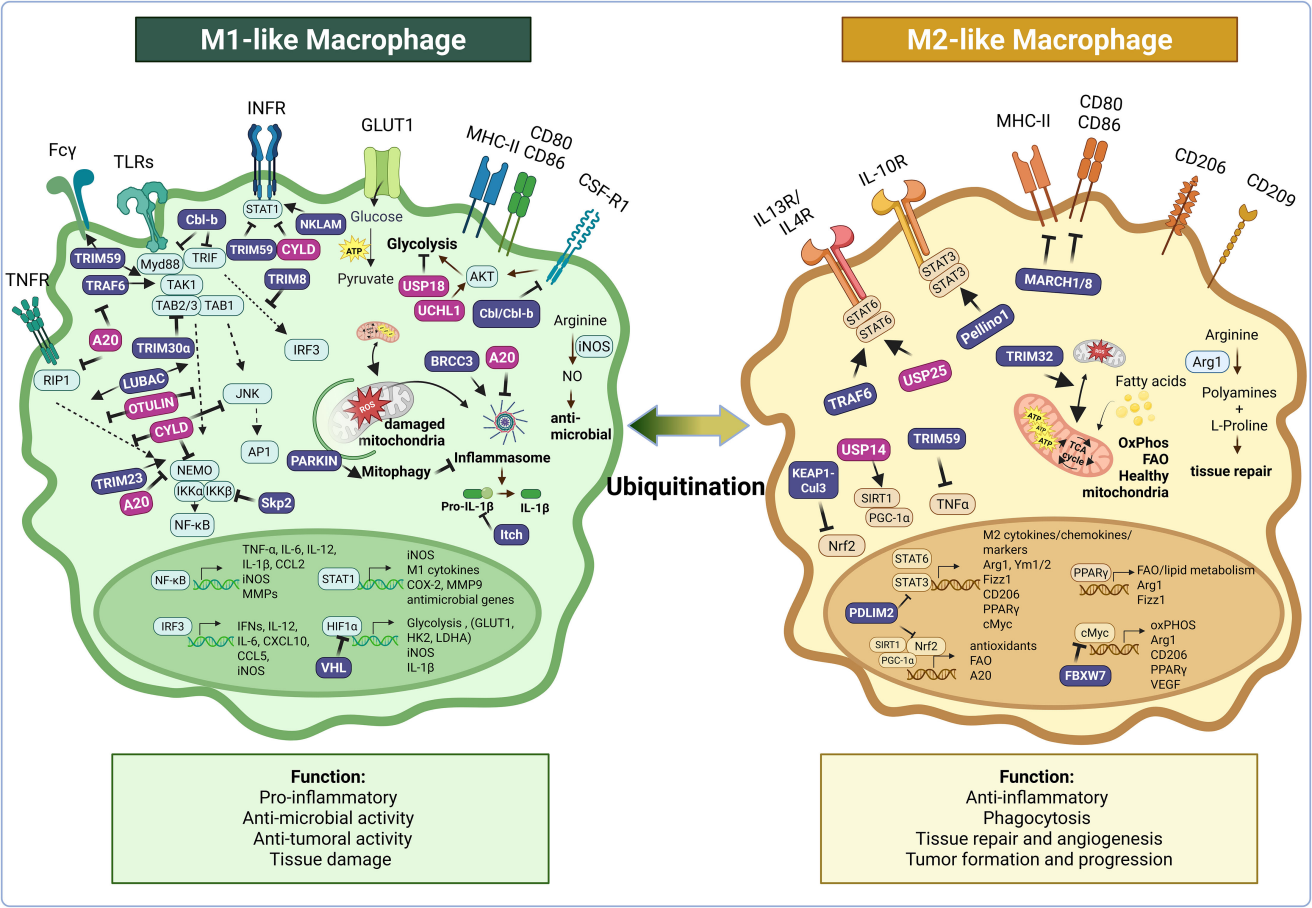
Ubiquitination plays a central role in regulating key signaling pathways that drive macrophage functional diversity and plasticity. This figure illustrates how numerous E3 ubiquitin ligases (in purple: Cbl-b, Cbl, TRIM8, TRIM23, TRIM30α, TRIM32, TRIM59, TRAF6, NKLAM, LUBAC, Skp2, VHL, Itch, BRCC3, Parkin, Keap1-Cul3, FBXW7, PDLIM2, Pellino1, MARCH1/8) and deubiquitinases (in pink: A20, CYLD, UCHL1, OTULIN, USP14, USP18, USP25) modulate the functional responses of pro-inflammatory M1-like and anti-inflammatory M2-like macrophages—as well as transitions between these states—by targeting key regulators of inflammatory and metabolic pathways for activation or degradation. Ubiquitination acts at multiple levels of the inflammatory cascade, controlling the expression or stability of receptors (e.g., CSF-1R, Fcγ, MHCII, CD80/86), inflammasome assembly, and the activation of proximal signaling cascades downstream of key inflammatory receptors (e.g., TNFR, IFNR, TLR, IL-10R, IL-13/IL-4R), as well as the nuclear translocation of key transcription factors (e.g., AP-1, NF-κB, STATs). It also regulates macrophage metabolism by modulating glycolysis, oxidative phosphorylation, hypoxia adaptation, fatty acid oxidation, arginine metabolism, and mitophagy. Arrows indicate positive regulation; blunt-end lines indicate inhibition. While this figure focuses on M1- and M2-like macrophages, where most mechanistic data related to ubiquitination are currently available, it is also known that ubiquitination regulates other functionally relevant macrophage states, such as foam cells and dying macrophages, which are not depicted here. Created in BioRender. Paolino, M. (2025) https://BioRender.com/i788nmb.

Classical M1 activation, leading to pro-inflammatory macrophages, is typically triggered by TLR ligands like LPS and cytokines such as IFN-γ, TNF-α, and IL-1 (9) (**Figure 2**). These signals converge on transcription factors NF-κB, AP-1, and IRFs, promoting the production of IL-12, IL-6, IL-1, TNF-α, and iNOS (10, 11). Ubiquitin signaling regulates these inflammatory pathways at multiple levels, from receptor activation to transcription factor control and negative feedback.

Upon TLR engagement, NLRX1 is rapidly ubiquitinated, dissociates from TRAF6, and then binds the IKK complex, resulting in inhibition of IKK phosphorylation and NF-κB activation (12). Similarly, the E3 ligase TRIM23 promotes NF-κB activation during viral infections by conjugating atypical Lysine 27-linked chains on NEMO, which is crucial for IRF3 and NF-κB activation downstream of viral sensors TLR3 and RIG-I/MDA-5 (13). Ubiquitination also regulates inflammasome assembly; the deubiquitinase BRCC3, as part of the BRISC complex, removes K48/K63 ubiquitin from NLRP3, permitting ASC oligomerization, caspase-1 activation, and IL-1β maturation (14). Loss of BRCC3 or activation of the vitamin D receptor (which inhibits BRCC3) blocks IL-1β release, highlighting the critical role of this deubiquitination step (15).

Additionally, both deubiquitinases and E3 ligases restrain M1 polarization to prevent excessive inflammation. A20 is a unique ubiquitin-editing enzyme with dual functions: it removes activating ubiquitin chains and adds degradative Lys48-linked chains to key adaptors in the NF-κB and MAPK pathways, including TRAF6, RIP1, and NEMO, thereby terminating signal transduction (16). A20 is also known to inhibit inflammasome activation by promoting the ubiquitination and degradation of NEK7 (17). A20-deficient macrophages exhibit prolonged activation and excessive cytokine production, leading to heightened inflammation (18). The deubiquitinase CYLD removes K63-linked chains from NF-κB (TRAF2/6, NEMO, RIP1) and JNK (TAK1) adaptors as well as STAT1, curbing M1 cytokine output and preventing inflammation from escalating into pathology (19). Consequently, CYLD-deficient macrophages exhibit hyperinflammatory signaling and are more susceptible to developing colitis-associated colorectal cancer (20). The deubiquitinase OTULIN adds further control by hydrolyzing linear, M1-linked ubiquitin chains on key adaptors of TLR and TNF signaling, which are linked by the E3 ligase complex LUBAC (21); OTULIN loss causes ligand-independent NF-κB activation and severe inflammation, which mimics the human OTULIN related autoinflammatory syndrome (ORAS) and is reversed by TNFα blockade (22).

The E3 ligases Cbl-b, Itch, and GRAIL, with broad effects as key negative regulators of immunity, dampen inflammatory signals linked to M1-like polarization by acting on key receptors and adaptors. Cbl-b ubiquitinates MyD88 and TRIF after CD11b–Src–Syk signaling, ending TLR signaling (23). Cblb^–/–^ macrophages overproduce cytokines upon TLR4 activation, with varying outcomes depending on infection: exaggerated cytokine responses in *T. gondii* infection (24) but improved survival in Candida sepsis (25). Itch restrains IL-1α amplification by indirectly promoting pro-IL-1α deubiquitination through yet unknown DUBs. Itch deficiency leads to hyper-M1 responses and worsens osteoarthritis, reversible with IL-1α neutralization (26, 27). GRAIL directly suppresses LPS‐driven M1 polarization by downregulating IL-1β, TNF-α, IL-6, and TLR4, limiting macrophage-driven tissue damage in models of endotoxemia as well as lung injury (28–30).

Other E3 ligases, like TRIM30α and TRIM8, are induced during inflammation as negative feedback mechanisms to terminate macrophage activation. TRIM30α targets the adaptors TAB2/TAB3 for degradation to shut off NF-κB; TRIM30α overexpression protects against endotoxin shock, while its loss prolongs cytokine release (31). TRIM8 ubiquitinates the adaptor TRIF, preventing binding to the TBK1 kinase and limiting IRF3 and NF-κB activation; its loss leads to cytokine overproduction in response to poly(I:C) or LPS (32). The E3 ligase Skp2, part of the SCF E3 ligase complex, promotes autophagic degradation of phosphorylated IKKβ via p62, helping resolve NF-κB signaling; without Skp2, NF-κB activation and cytokine output persist (33).

Ubiquitin also regulates M1-linked transcription factors. The nuclear E3 ligase PDLIM2 ubiquitinates NF-κB p65 (RelA) and STAT3, restraining inflammation and blocking M2 skewing via IL-10. In tumors, oxidative stress suppresses PDLIM2, impairing macrophage anti-tumor responses; PDLIM2 loss drives unchecked STAT3 activity and pro-tumor M2 skewing, while restoration improves anti-tumor function (34). Similarly, other E3 ligases play crucial roles in macrophage polarization and function in tissue injury and disease contexts. For instance, TRIM21 expression promotes M1 polarization post-myocardial infarction; TRIM21-deficient mice exhibit smaller infarcts, better cardiac function, and increased M2 macrophages (35). TRIM59 enhances bactericidal M1 functions during sepsis; it ubiquitinates Fcγ receptor machinery and NF-κB adaptors to sustain antimicrobial activity and control inflammation. Its deletion impairs Fcγ receptor expression and phagocytosis, exaggerating cytokine production (36).

### 2.2 Immunosuppressive M2-like polarization

M2-like macrophages typically arise in response to IL-4/IL-13 via STAT6, or IL-10 and glucocorticoids via STAT3. Characteristically, they express high levels of Arg1, Ym1, Fizz1, IL-10, and scavenger receptors like CD206. Functionally, they promote tissue repair, fibrosis, and, importantly, support tumor progression (**Figure 2**). The ubiquitin system is equally crucial in driving and fine-tuning these anti-inflammatory programs (37).

IL-10–induced M2 polarization depends on ubiquitination. The E3 ligase Pellino1 enhances STAT3 stability and in turn its activity by mediating its ubiquitination. Pellino1 levels are elevated in mice and humans with colitis, and if depleted from monocytes it reduces colon inflammation and colorectal cancer (38). IL-10 induces MARCH1, an E3 ligase that ubiquitinates MHC II and CD86, targeting them for degradation. This strips macrophages of antigen-presenting functions, enforcing an immunosuppressive M2 state. In March1^-/-^ cells, IL-10 fails to reduce MHC II and CD86, preserving T cell activation and inflammation (39). TRIM59, another E3 ligase, which limits M1 polarization by ubiquitinating and degrading STAT1 (40), is induced by IL-4/IL-13 and helps maintain M2 identity. Though not essential for canonical M2 marker expression, TRIM59 restrains aberrant TNF-α production. Without TRIM59, M2 macrophages overproduce TNF-α, enhancing tumor invasion via TNF-responsive genes like MMP-9. Blocking TNF-α or downstream genes reverses this, showing that TRIM59 preserves M2’s anti-inflammatory, tumor-supportive role (41).

Ubiquitination also constrains M2 polarization. For instance, the E3 ligase FBXW7 suppresses the tumor-promoting M2 program in tumor-associated macrophages by targeting c-Myc (42). The E3 ligase PDLIM2 inhibits IL-10–driven M2 polarization by promoting STAT3 degradation. When PDLIM2 is repressed (e.g., by reactive oxygen species -ROS-mediated activation of the transcription factor BACH1 in tumors), STAT3 persists, pushing macrophages into a highly suppressive state. Restoring PDLIM2 reactivates this degradation switch, limiting fibrosis and the immunosuppressive tumor microenvironment (34).

Ubiquitin-modifying enzymes active in M1 signaling also support M2 polarization, highlighting the versatility of ubiquitin regulators. Both the E3 ligase TRAF3 and the deubiquitinase USP25 converge on preserving STAT6 activity. TRAF3 binds STAT6 and adds K63-linked ubiquitin, protecting it from degradation and boosting M2 genes like Arg1, Ym1, and Fizz1. Similarly, USP25 removes K48-linked ubiquitin from STAT6, stabilizing it. USP25-deficient macrophages show impaired M2 gene expression, and mice lacking USP25 are protected from M2-driven fibrosis due to reduced macrophage accumulation. Finally, ubiquitination events that limit M1 signaling can simultaneously promote M2 responses (43, 44). A key example is A20, which as described above, shuts down NF-κB and inflammasome signaling, allowing IL-4/STAT6 signaling. In myeloid-specific A20 knockout mice, macrophages skew toward M1 and fail to mount type 2 responses during helminth infection (18).

## 3 Ubiquitin regulation of metabolic reprogramming in macrophages

Metabolic reprogramming is a hallmark of macrophage activation. While it is most well described for canonical M1 and M2-like phenotypes, reprogramming to meet specific energy requirements is believed to occur across all dynamic polarization states, including those not covered in this review. Pro-inflammatory M1-like polarization shifts metabolism toward glycolysis and disrupts the tricarboxylic acid cycle (6), whereas the anti-inflammatory and repair functions of M2-like macrophages rely on mitochondrial respiration and fatty acid oxidation (5, 7). The ubiquitin system critically influences macrophage metabolism and phenotype through various mechanisms, helping to integrate metabolic and inflammatory signals to fine tune immune outcomes (**Figure 2**).

### 3.1 Glycolysis and hypoxic adaptations

A key driver of glycolytic reprogramming in M1 macrophages is the transcription factor HIF-1α, which induces glycolytic enzymes and supports IL-1β production (45). The E3 ligase VHL controls HIF-1α to restrain the M1 glycolytic burst required for inflammatory activation. Under normoxia, VHL ubiquitinates HIF-1α for degradation, limiting glycolysis. In hypoxia or VHL absence, HIF-1α stabilizes, promoting glycolysis and inflammatory gene expression (46). Myeloid cells lacking VHL or with constitutively active HIF-1α show enhanced glycolysis and a hyper-inflammatory profile.

Ubiquitin also regulates growth factor signaling linked to metabolism. The RING-type E3 ligases Cbl and Cbl-b target the colony-stimulating factor receptor (CSF-1R) for trafficking into macropinosomes, leading to its degradation. Without Cbl/Cbl-b, CSF-1R signaling is prolonged, leading to sustained AKT activation, elevated inflammatory gene expression, and increased proliferation (47). This links Cbl/Cbl-b loss to heightened glycolytic and inflammatory states, acting as a brake on growth factor-driven metabolic activation in M1-like macrophages.

The PI3K/AKT pathway is another key metabolic axis influenced by ubiquitination. The deubiquitinase UCHL1 promotes M1 polarization by enhancing AKT signaling; not by deubiquitinating AKT, but by facilitating autophagic degradation of the PI3K p110α subunit. This optimizes the signaling output and increases AKT phosphorylation, skewing macrophages toward a glycolytic, M1 phenotype (48). Uchl1^–/–^ macrophages show reduced AKT activity and lower M1 markers like iNOS and TNF-α, supporting UCHL1’s role in sustaining metabolic signaling for M1 activation.

Conversely, other DUBs inhibit glycolytic metabolism and inflammation. USP18, induced by type I interferons, redirects metabolism away from glycolysis and cytokine production, promoting oxidative metabolism typical of M2 macrophages (49). USP18-deficient cells remain locked in a glycolytic, pro-inflammatory state, even under anti-inflammatory conditions, highlighting USP18 as a switch between M1 and M2 metabolic states.

### 3.2 Lipid metabolism

Macrophage polarization is closely linked to lipid metabolism (50). M1 macrophages accumulate lipids, while M2 macrophages preferentially oxidize fatty acids. E3 ubiquitin ligases regulate systemic lipid metabolism and inflammation, as shown in obesity models. Mice lacking the E3 ligase Itch are protected from diet-induced obesity and insulin resistance, with adipose tissue macrophages shifting to an M2-like state (51). These macrophages express higher M2 markers like Arg1 and CD206, along with fatty acid oxidation genes, and produce fewer pro-inflammatory cytokines, suggesting Itch limits the oxidative M2 program. Conversely, Cbl-b plays a protective role in metabolic syndrome by restraining lipid-driven inflammation. In diet-induced obesity, Cbl-b^–/–^ mice show worsened insulin resistance due to heightened macrophage activation in adipose tissue (52). Cbl-b ubiquitinates TLR4 in macrophages, promoting its downregulation. Without Cbl-b, prolonged TLR4 signaling leads to excessive IL-6 and TNF-α production, driving chronic inflammation and insulin resistance. In atherosclerosis models, Cbl-b deficiency worsens plaque formation by increasing pro-inflammatory macrophage accumulation (53). Thus, Cbl-b acts as a lipid-sensing checkpoint, preventing macrophage overactivation and preserving metabolic homeostasis.

Ubiquitin pathways also interact with nuclear receptors and transcription factors regulating lipid metabolism in macrophages. PPARγ, a lipid-activated transcription factor, is essential for the M2 phenotype, promoting fatty acid uptake and oxidation (54). The deubiquitinase USP25 promotes IL-4–elicited M2 polarization by preserving STAT6 levels, allowing full induction of PPAR-γ, and in turn, the expression of M2-associated genes including those involved in lipid metabolism. In USP25^-/-^ BMDMs, STAT6 activation is blunted, leading to reduced PPARγ expression and impaired M2 polarization. USP25^-/-^ macrophages also exhibit defective IL-4-driven polarization, lower expression of M2 markers, and fatty acid metabolic enzymes. Consequently, USP25^-/-^ mice are protected from fibrosis in M2-associated models (43). USP14 promotes M2 macrophage polarization by stabilizing the transcription factor SIRT1 through deubiquitination. SIRT1, together with the coactivator PGC-1α, drives the expression of genes involved in fatty acid oxidation (55). These findings highlight the importance of ubiquitin-mediated stabilization of lipid metabolism regulators in M2 metabolic reprogramming.

Macrophages play a key role in cholesterol and lipoprotein homeostasis by taking up cholesterol-rich lipoproteins and promoting reverse cholesterol transport to the liver for excretion. In atherosclerosis, monocyte-derived macrophages take up oxidized low-density lipoproteins (oxLDL) deposited in the arterial walls *via* scavenger receptors such as CD36 or SR-B1 and become lipid-laden foam cells—a key event in the onset and progression of atherosclerosis that is tightly regulated by ubiquitination (56). For example, the deubiquitinase UCHL1 stabilizes CD36 by removing K48-linked ubiquitin, promoting oxLDL uptake and foam cell formation; inhibiting UCHL1 leads to CD36 degradation and reduced lipid accumulation (57). In contrast, the transmembrane E3 ligase GRAIL enhances foam cell formation by catalyzing K63-linked ubiquitination of SR-B1, increasing its recycling to the macrophage surface and boosting oxLDL uptake (58). For cholesterol efflux, cholesterol-rich conditions trigger the ubiquitination and lysosomal degradation of the cholesterol efflux transporter ABCA1 (59), and silencing of the E3 ligase HECTD1 increases ABCA1 levels and enhances cholesterol efflux (60), further underscoring the role of ubiquitination in regulating foam cell dynamics. Liver X Receptors (LXRs), key cholesterol-sensing nuclear receptors that coordinate lipid metabolism and inflammation in macrophages (61), induce efflux transporters (ABCA1, ABCG1) and repress inflammatory genes in cholesterol-loaded cells (62). LXRs also drive the expression of E3 ligases such as IDO and RNF145, which ubiquitinate the LDL receptor (63) and HMG-CoA reductase (64), respectively, targeting them for degradation. This limits cholesterol uptake and links LXR activity to sterol biosynthesis. LXRα is itself subject to ubiquitin-dependent regulation: in the absence of ligand, it is targeted for degradation by the BRCA1–BARD1 E3 ubiquitin ligase complex, whereas ligand binding prevents this, stabilizing LXRα and sustaining its transcriptional activity (65).

### 3.3 Mitochondrial function and oxidative stress

Mitochondrial function and quality control are vital to macrophage immunometabolism. M1 macrophages accumulate damaged mitochondria and ROS, triggering inflammasome activation. In contrast, M2 macrophages maintain healthy mitochondria for efficient oxidative phosphorylation (66). Ubiquitin-dependent mitophagy, mediated by the E3 ligase Parkin and kinase PINK1, clears damaged mitochondria. In Parkin^–/–^ or Pink1^–/–^ macrophages, where mitophagy is impaired, dysfunctional mitochondria accumulate, shifting metabolism toward oxidative metabolism and M2-like features. These macrophages fail to induce glycolysis and nitric oxide production typical of the M1 response but still produce M1-like inflammatory cytokines such as IL-1β (67). Parkin deficiency exacerbates inflammation through unchecked inflammasome activation, as mitophagy disruption impairs induction of A20, a negative regulator of NF-κB and inflammasome signaling. Without A20, NF-κB-driven cytokines like IL-1β and NLRP3 are overproduced, driving inflammation and fibrosis, highlighting mitophagy’s role in preventing excessive inflammatory responses linked to mitochondrial dysfunction (68).

The Keap1–Nrf2 pathway links ubiquitin signaling to redox balance, promoting the anti-inflammatory response typically associated with M2 macrophage polarization. Under normal conditions, Keap1, a substrate adaptor for the Cul3-RING E3 ligase, guides ubiquitinates Nrf2 for degradation. However, upon oxidative stress, Keap1’s function is impaired, allowing Nrf2 to escape degradation, translocate to the nucleus, and induce antioxidant genes that protect mitochondria (69). Additionally, Nrf2 promotes A20 expression, which dampens NF-κB and IL-1β output (70). Thus, Keap1 acts as a gatekeeper for oxidative metabolism and anti-inflammatory tone.

Autophagy, essential to cellular metabolism, is regulated by ubiquitination to support macrophage fitness during infection. In Mycobacterium tuberculosis infection, TRIM32, a tripartite-motif E3 ligase, facilitates autophagic degradation of bacteria and damaged mitochondria. TRIM32-deficient macrophages show impaired LC3 recruitment and bacterial clearance, while TRIM32 overexpression enhances autophagic flux and preserves mitochondrial health under stress (71).

### 3.4 Amino acid metabolism

Amino acid metabolism, especially L-arginine utilization, distinguishes M1 and M2 macrophages. M1 macrophages produce and use iNOS to convert arginine into nitric oxide -NO- for microbial killing, while M2 macrophages express arginase 1 -Arg1- to produce ornithine which is then converted into polyamines and L-proline for tissue repair. Ubiquitination also regulates this metabolic divergence.

The E3 ligase RNF19b (NKLAM), induced by LPS/IFN-γ, enhances iNOS expression and NO production in M1 macrophages by inducing STAT1 and NF-κB signaling. Nklam^-/-^ macrophages show reduced NO production and iNOS expression, with attenuated STAT1 Tyr701 phosphorylation and delayed NF-κB p65 nuclear entry, leading to lower NF-κB activity and impaired NO−dependent bacterial killing (72). Thus, NKLAM supports M1 polarization and NO-mediated microbicidal function.

Conversely, ubiquitin pathways that limit Arg1 levels can restrain M2 metabolism. In tumor associated macrophages, c-Myc drives Arg1^high^ and IL-10^high^ states. The F−box protein FBXW7, part of an SCF E3 ligase, targets c-Myc for degradation. FBXW7 loss stabilizes c-Myc, hyperactivating M2 genes and increasing CD206^+^ macrophages and tumor growth. Hence, FBXW7 limits the pro-tumoral, Arg1-driven M2 phenotype (42). Ubiquitination also controls Arg1 induction by regulating IL-4/STAT6. The E3 ligase TRAF3 and the deubiquitinase USP25, by stabilizing IL-4/STAT6, as mechanistically explained above, promote the expression of Arg1 and other M2 genes. Without TRAF3 or USP25, IL-4-induced Arg1 is impaired, undermining M2 polarization (43). Lastly, TRIM14 modulates iNOS activity to optimize antimicrobial defense; Trim14^-/-^ macrophages show elevated iNOS activity resulting in heightened NO and stronger bacterial control (73).

## 4 Ubiquitination and macrophage cell death pathways

Although not the focus of this review, it is worth noting that ubiquitination also regulates macrophage cell death pathways, including apoptosis, necroptosis and ferroptosis. For example, the deubiquitinating enzyme USP22 has been shown to protect macrophages from apoptosis under pro-inflammatory conditions by stabilizing pro-survival factors (74). In contrast, the E3 ligase TRIM25 can promote cell death by driving necroptosis of oxidized LDL-challenged macrophages by ubiquitinating the DNA repair factor XRCC1, thereby triggering PARP1- and RIPK3-dependent cell death (75). In ferroptosis, the Keap1–Cullin-3 E3 ligase complex normally ubiquitinates the transcription factor Nrf2, targeting it for proteasomal degradation (76). When this brake is lifted, Nrf2 accumulates and induces anti-ferroptotic genes such as SLC7A11 and GPX4, which protect macrophages from lipid peroxidation-induced death (77). During *T. gondii* infections, the parasite dense granule protein GRA35 interacts with the host E3 ligase Itch to promote NLRP1 inflammasome–dependent macrophage pyroptosis, a process that is blocked by proteasome inhibition or loss of Itch (78).

## 5 Discussion

Throughout this review, we have highlighted ubiquitination as a crucial regulator of macrophage biology, precisely modulating immunometabolism and polarization. Through the action of E3 ubiquitin ligases and deubiquitinases, ubiquitination integrates key metabolic regulators of glucose, lipid, mitochondrial, and amino acid metabolism with major polarization signaling pathways, enabling macrophages to adapt their functions to various immune and metabolic cues. Dysregulation of these pathways can lead to pathogenesis, underscoring their importance. Loss of negative regulators like A20, CYLD, or OTULIN results in unchecked M1 polarization, causing excessive inflammation and tissue damage that contribute to autoinflammatory diseases or cancer (20, 22). Conversely, an overactive M2 program promotes tumor-associated macrophages, aiding immune evasion and tumor progression. Ubiquitin ligases such as FBXW7, PDLIM2, and GRAIL act as brakes on M2 polarization, and their loss can worsen cancer outcomes (34, 42).

This knowledge opens therapeutic possibilities for macrophage reprogramming in disease settings. Enhancing negative regulators like Cbl-b or GRAIL could reduce inflammation in conditions like septic shock or cytokine storms. GRAIL overexpression protects against LPS-induced acute lung injury by downregulating TLR4. High GRAIL expression in lung cancer correlates with improved survival, suggesting enhanced ubiquitin regulation can control tumor-promoting inflammation (28). Conversely, inhibiting E3 ligases or DUBs could reawaken macrophage inflammatory activity in tumors. For example, deleting USP18 in tumor-associated macrophages activates type I interferon responses, depleting M2 TAMs and slowing tumor growth (79). Additionally, promoting MAEA activity to enhance macrophage phagocytosis has been shown to inhibit tumor progression in preclinical models (80).

Future research to map the full range of ubiquitin substrates and to understand the context-dependent roles of E3 ligases and DUBs in macrophages is essential. Many ubiquitin-modulating enzymes likely have novel or additional, yet unexplored, targets in macrophages. Expanding this knowledge could open new therapeutic opportunities. While strategies targeting macrophage metabolism with nutrients, nanoparticles, or small molecules show promise (81), those that focus on core pathways like glycolysis or fatty acid oxidation often exert broad, off-target effects For example, common metabolic inhibitors like 2-deoxy-D-glucose and etomoxir are known to affect multiple aspects of macrophage metabolism (altering ATP production, signaling pathways, CoA levels, etc.) (82). These overlapping roles and off-target effects make it difficult to achieve precise, disease‐specific modulation of macrophage metabolism (83). In contrast, targeting ubiquitin enzymes offers a more direct and finely tuned approach to modulate macrophage functions, providing greater specificity for therapeutic interventions. The rapid development of tools to modulate ubiquitination holds significant potential for manipulating macrophage pathways. For instance, using targeted drugs, such as small-molecule inhibitors, to block a DUB or E3 ligase that supports immunosuppressive pathways could trigger a strong anti-tumor macrophage response, while designing PROTACs to degrade key ubiquitin regulators of inflammation could reduce macrophage-mediated tissue damage in infections and autoimmune diseases.

In conclusion, ubiquitination acts as a master regulator of macrophage function, controlling their plasticity to adapt to diverse environmental and metabolic challenges. Harnessing the ubiquitin system to modulate macrophage inflammatory and immunometabolic pathways—and consequently their polarization—offers a promising strategy for developing targeted therapies to either temper inflammation or enhance macrophage functions in infections and cancer.

## References

[1] Damgaard RB. The ubiquitin system: from cell signalling to disease biology and new therapeutic opportunities. Cell Death Differ. (2021) 28:423–6. doi: 10.1038/s41418-020-00703-w, PMID: 33446876 PMC7862391

[2] Sewduth RN Baietti MF Sablina AA. Cracking the monoubiquitin code of genetic diseases. IJMS. (2020) 21:3036. doi: 10.3390/ijms21093036, PMID: 32344852 PMC7246618

[3] Komander D Rape M. The ubiquitin code. Annu Rev Biochem. (2012) 81:203–29. doi: 10.1146/annurev-biochem-060310-170328, PMID: 22524316

[4] Chen J Chen ZJ. Regulation of NF-κB by ubiquitination. Curr Opin Immunol. (2013) 25:4–12. doi: 10.1016/j.coi.2012.12.005, PMID: 23312890 PMC3594545

[5] Chen S Saeed AFUH Liu Q Jiang Q Xu H Xiao GG. Macrophages in immunoregulation and therapeutics. Signal Transduct Target Ther. (2023) 8:1–35. doi: 10.1038/s41392-023-01452-1, PMID: 37211559 PMC10200802

[6] Soto-Heredero G Gómez de las Heras MM Gabandé-Rodríguez E Oller J Mittelbrunn M. Glycolysis - a key player in the inflammatory response. FEBS J. (2020) 287:3350–69. doi: 10.1111/febs.15327, PMID: 32255251 PMC7496292

[7] Liu Y Xu R Gu H Zhang E Qu J Cao W. Metabolic reprogramming in macrophage responses. biomark Res. (2021) 9:1. doi: 10.1186/s40364-020-00251-y, PMID: 33407885 PMC7786975

[8] Pérez S Rius-Pérez S. Macrophage polarization and reprogramming in acute inflammation: A redox perspective. Antioxid (Basel). (2022) 11:1394. doi: 10.3390/antiox11071394, PMID: 35883885 PMC9311967

[9] Müller E Christopoulos PF Halder S Lunde A Beraki K Speth M. Toll-like receptor ligands and interferon-γ Synergize for induction of antitumor M1 macrophages. Front Immunol. (2017) 8:1383. doi: 10.3389/fimmu.2017.01383, PMID: 29123526 PMC5662546

[10] Wang H Wang X Li X Fan Y Li G Guo C. CD68(+)HLA-DR(+) M1-like macrophages promote motility of HCC cells via NF-κB/FAK pathway. Cancer Lett. (2014) 345:91–9. doi: 10.1016/j.canlet.2013.11.013, PMID: 24333724

[11] Krausgruber T Blazek K Smallie T Alzabin S Lockstone H Sahgal N. IRF5 promotes inflammatory macrophage polarization and TH1-TH17 responses. Nat Immunol. (2011) 12:231–8. doi: 10.1038/ni.1990, PMID: 21240265

[12] Xia X Cui J Wang HY Zhu L Matsueda S Wang Q. NLRX1 negatively regulates TLR-induced NF-κB signaling by targeting TRAF6 and IKK. Immunity. (2011) 34:843–53. doi: 10.1016/j.immuni.2011.02.022, PMID: 21703539 PMC3150212

[13] Arimoto K Funami K Saeki Y Tanaka K Okawa K Takeuchi O. Polyubiquitin conjugation to NEMO by triparite motif protein 23 (TRIM23) is critical in antiviral defense. Proc Natl Acad Sci USA. (2010) 107:15856–61. doi: 10.1073/pnas.1004621107, PMID: 20724660 PMC2936632

[14] Py BF Kim M-S Vakifahmetoglu-Norberg H Yuan J. Deubiquitination of NLRP3 by BRCC3 critically regulates inflammasome activity. Mol Cell. (2013) 49:331–8. doi: 10.1016/j.molcel.2012.11.009, PMID: 23246432

[15] Rao Z Chen X Wu J Xiao M Zhang J Wang B. Vitamin D receptor inhibits NLRP3 activation by impeding its BRCC3-mediated deubiquitination. Front Immunol. (2019) 10:2783. doi: 10.3389/fimmu.2019.02783, PMID: 31866999 PMC6904361

[16] Polykratis A Martens A Eren RO Shirasaki Y Yamagishi M Yamaguchi Y. A20 prevents inflammasome-dependent arthritis by inhibiting macrophage necroptosis through its ZnF7 ubiquitin-binding domain. Nat Cell Biol. (2019) 21:731–42. doi: 10.1038/s41556-019-0324-3, PMID: 31086261

[17] Yu J Li H Wu Y Luo M Chen S Shen G. Inhibition of NLRP3 inflammasome activation by A20 through modulation of NEK7. Proc Natl Acad Sci U.S.A. (2024) 121:e2316551121. doi: 10.1073/pnas.2316551121, PMID: 38865260 PMC11194493

[18] Petta I Thorp M Ciers M Blancke G Boon L Meese T. Myeloid A20 is critical for alternative macrophage polarization and type-2 immune-mediated helminth resistance. Front Immunol. (2024) 15:1373745. doi: 10.3389/fimmu.2024.1373745, PMID: 38680500 PMC11045979

[19] Huyen NT Ngoc NT Giang NH Trang DT Hanh HH Binh VD. CYLD stimulates macrophage phagocytosis of leukemic cells through STAT1 signalling in acute myeloid leukemia. PloS One. (2023) 18:e0283586. doi: 10.1371/journal.pone.0283586, PMID: 37549179 PMC10406188

[20] Zhang J Stirling B Temmerman ST Ma CA Fuss IJ Derry JMJ. Impaired regulation of NF-κB and increased susceptibility to colitis-associated tumorigenesis in CYLD-deficient mice. J Clin Invest. (2006) 116:3042–9. doi: 10.1172/JCI28746, PMID: 17053834 PMC1616194

[21] Keusekotten K Elliott PR Glockner L Fiil BK Damgaard RB Kulathu Y. OTULIN antagonizes LUBAC signaling by specifically hydrolyzing Met1-linked polyubiquitin. Cell. (2013) 153:1312–26. doi: 10.1016/j.cell.2013.05.014, PMID: 23746843 PMC3690481

[22] Damgaard RB Walker JA Marco-Casanova P Morgan NV Titheradge HL Elliott PR. The deubiquitinase OTULIN is an essential negative regulator of inflammation and autoimmunity. Cell. (2016) 166:1215–1230.e20. doi: 10.1016/j.cell.2016.07.019, PMID: 27523608 PMC5002269

[23] Han C Jin J Xu S Liu H Li N Cao X. Integrin CD11b negatively regulates TLR-triggered inflammatory responses by activating Syk and promoting degradation of MyD88 and TRIF via Cbl-b. Nat Immunol. (2010) 11:734–42. doi: 10.1038/ni.1908, PMID: 20639876

[24] Wei H Wu S Mai L Yang L Zou W Peng H. Cbl-b negatively regulates TLR/MyD88-mediated anti- Toxoplasma gondii immunity. Microbiol Spectr. (2023) 11:e00074–23. doi: 10.1128/spectrum.00074-23, PMID: 37909781 PMC10714978

[25] Wirnsberger G Zwolanek F Asaoka T Kozieradzki I Tortola L Wimmer RA. Inhibition of CBLB protects from lethal Candida albicans sepsis. Nat Med. (2016) 22:915–23. doi: 10.1038/nm.4134, PMID: 27428901 PMC6209141

[26] Lin X Wang W McDavid A Xu H Boyce BF Xing L. The E3 ubiquitin ligase Itch limits the progression of post-traumatic osteoarthritis in mice by inhibiting macrophage polarization. Osteoarthr Cartil. (2021) 29:1225–36. doi: 10.1016/j.joca.2021.04.009, PMID: 33940137 PMC8319075

[27] Lin X Zhang H Boyce BF Xing L. Ubiquitination of interleukin-1α is associated with increased pro-inflammatory polarization of murine macrophages deficient in the E3 ligase ITCH. J Biol Chem. (2020) 295:11764–75. doi: 10.1074/jbc.RA120.014298, PMID: 32587089 PMC7450106

[28] Fujimoto S Kyuichi K Kaede Y Chihiro Y Emi I Ryo I. RNF128 expression in lung adenocarcinoma is a favorable prognostic factor associated with decreased tumor-associated macrophages. Thorac Cancer. (2023) 14:1581–8. doi: 10.1111/1759-7714.14901, PMID: 37186218 PMC10260493

[29] Shih C-C Liu P-Y Chen J-H Liao M-H Hsieh C-M Ka S-M. Macrophage expression of E3 ubiquitin ligase Grail protects mice from lipopolysaccharide-induced hyperinflammation and organ injury. PloS One. (2018) 13:e0208279. doi: 10.1371/journal.pone.0208279, PMID: 30571701 PMC6301572

[30] Liu P-Y Chen C-Y Lin Y-L Lin C-M Tsai W-C Tsai Y-L. RNF128 regulates neutrophil infiltration and myeloperoxidase functions to prevent acute lung injury. Cell Death Dis. (2023) 14:369. doi: 10.1038/s41419-023-05890-1, PMID: 37344492 PMC10284794

[31] Shi M Deng W Bi E Mao K Ji Y Lin G. TRIM30α negatively regulates TLR-mediated NF-κB activation by targeting TAB2 and TAB3 for degradation. Nat Immunol. (2008) 9:369–77. doi: 10.1038/ni1577, PMID: 18345001

[32] Ye W Hu M-M Lei C-Q Zhou Q Lin H Sun M-S. TRIM8 negatively regulates TLR3/4-mediated innate immune response by blocking TRIF–TBK1 interaction. J Immunol. (2017) 199:1856–64. doi: 10.4049/jimmunol.1601647, PMID: 28747347

[33] Liu K Zhang L Zhao Q Zhao Z Zhi F Qin Y. SKP2 attenuates NF-κB signaling by mediating IKKβ degradation through autophagy. J Mol Cell Biol. (2018) 10:205–15. doi: 10.1093/jmcb/mjy012, PMID: 29474632

[34] Li L Sun F Han L Liu X Xiao Y Gregory AD. PDLIM2 repression by ROS in alveolar macrophages promotes lung tumorigenesis. JCI Insight. (2021) 6:e144394. doi: 10.1172/jci.insight.144394, PMID: 33539325 PMC8021114

[35] Li Z Liu X Zhang X Zhang W Gong M Qin X. TRIM21 aggravates cardiac injury after myocardial infarction by promoting M1 macrophage polarization. Front Immunol. (2022) 13:1053171. doi: 10.3389/fimmu.2022.1053171, PMID: 36439111 PMC9684192

[36] Jin Z Zhu Z Liu S Hou Y Tang M Zhu P. TRIM59 protects mice from sepsis by regulating inflammation and phagocytosis in macrophages. Front Immunol. (2020) 11:263. doi: 10.3389/fimmu.2020.00263, PMID: 32133014 PMC7041419

[37] Rőszer T. Understanding the mysterious M2 macrophage through activation markers and effector mechanisms. Mediators Inflammation. (2015) 2015:816460. doi: 10.1155/2015/816460, PMID: 26089604 PMC4452191

[38] Hwang S Park J Koo S-Y Lee S-Y Jo Y Ryu D. The ubiquitin ligase Pellino1 targets STAT3 to regulate macrophage-mediated inflammation and tumor development. Nat Commun. (2025) 16:1256. doi: 10.1038/s41467-025-56440-6, PMID: 39893188 PMC11787384

[39] Galbas T Raymond M Sabourin A Bourgeois-Daigneault M-C Guimont-Desrochers F Yun TJ. MARCH1 E3 ubiquitin ligase dampens the innate inflammatory response by modulating monocyte functions in mice. J Immunol. (2017) 198:852–61. doi: 10.4049/jimmunol.1601168, PMID: 27940660

[40] Wang H Lou J Liu H Liu Y Xie B Zhang W. TRIM59 deficiency promotes M1 macrophage activation and inhibits colorectal cancer through the STAT1 signaling pathway. Sci Rep. (2024) 14:16081. doi: 10.1038/s41598-024-66388-0, PMID: 38992114 PMC11239810

[41] Tian Y Guo Y Zhu P Zhang D Liu S Tang M. TRIM59 loss in M2 macrophages promotes melanoma migration and invasion by upregulating MMP-9 and Madcam1. Aging. (2019) 11:8623–41. doi: 10.18632/aging.102351, PMID: 31600735 PMC6814609

[42] Zhong L Zhang Y Li M Song Y Liu D Yang X. E3 ligase FBXW7 restricts M2-like tumor-associated macrophage polarization by targeting c-Myc. Aging. (2020) 12:24394–423. doi: 10.18632/aging.202293, PMID: 33260160 PMC7762499

[43] Xu Y Liu J Wang J Wang J Lan P Wang T. USP25 stabilizes STAT6 to promote IL-4-induced macrophage M2 polarization and fibrosis. Int J Biol Sci. (2025) 21:475–89. doi: 10.7150/ijbs.99345, PMID: 39781451 PMC11705635

[44] Shi J-H Liu L-N Song D-D Liu W-W Ling C Wu F-X. TRAF3/STAT6 axis regulates macrophage polarization and tumor progression. Cell Death Differ. (2023) 30:2005–16. doi: 10.1038/s41418-023-01194-1, PMID: 37474750 PMC10406838

[45] Corcoran SE O’Neill LAJ. HIF1α and metabolic reprogramming in inflammation. J Clin Invest. (2016) 126:3699–707. doi: 10.1172/JCI84431, PMID: 27571407 PMC5096812

[46] Cramer T Yamanishi Y Clausen BE Förster I Pawlinski R Mackman N. HIF-1α Is essential for myeloid cell-mediated inflammation. Cell. (2003) 112:645–57. doi: 10.1016/S0092-8674(03)00154-5, PMID: 12628185 PMC4480774

[47] Huang L Thiex NW Lou J Ahmad G An W Low-Nam ST. The ubiquitin ligases Cbl and Cbl-b regulate macrophage growth by controlling CSF-1R import into macropinosomes. MBoC. (2024) 35:ar38. doi: 10.1091/mbc.E23-09-0345, PMID: 38170572 PMC10916879

[48] Huang Y He S Chen Y Sheng J Fu Y Du X. UCHL1 promoted polarization of M1 macrophages by regulating the PI3K/AKT signaling pathway. JIR. (2022) 15:735–46. doi: 10.2147/JIR.S343487, PMID: 35153498 PMC8824699

[49] Lai J-H Wu D-W Huang C-Y Hung L-F Wu C-H Ho L-J. USP18 induction regulates immunometabolism to attenuate M1 signal-polarized macrophages and enhance IL-4-polarized macrophages in systemic lupus erythematosus. Clin Immunol. (2024) 265:110285. doi: 10.1016/j.clim.2024.110285, PMID: 38880201

[50] Batista-Gonzalez A Vidal R Criollo A Carreño LJ. New insights on the role of lipid metabolism in the metabolic reprogramming of macrophages. Front Immunol. (2020) doi:2993. doi: 10.3389/fimmu.2019.02993, PMID: 31998297 PMC6966486

[51] Marino A Menghini R Fabrizi M Casagrande V Mavilio M Stoehr R. ITCH deficiency protects from diet-induced obesity. Diabetes. (2014) 63:550–61. doi: 10.2337/db13-0802, PMID: 24170694

[52] Abe T Hirasaka K Kagawa S Kohno S Ochi A Utsunomiya K. Cbl-b is a critical regulator of macrophage activation associated with obesity-induced insulin resistance in mice. Diabetes. (2013) 62:1957–69. doi: 10.2337/db12-0677, PMID: 23349502 PMC3661636

[53] Seijkens TTP Poels K Meiler S Van Tiel CM Kusters PJH Reiche M. Deficiency of the T cell regulator Casitas B-cell lymphoma-B aggravates atherosclerosis by inducing CD8+ T cell-mediated macrophage death. Eur Heart J. (2019) 40:372–82. doi: 10.1093/eurheartj/ehy714, PMID: 30452556 PMC6340101

[54] Vogel A Brunner JS Hajto A Sharif O Schabbauer G. Lipid scavenging macrophages and inflammation. Biochim Biophys Acta (BBA) - Mol Cell Biol Lipids. (2022) 1867:159066. doi: 10.1016/j.bbalip.2021.159066, PMID: 34626791

[55] He F Chen Y He D He S. USP14-mediated deubiquitination of SIRT1 in macrophage promotes fatty acid oxidation amplification and M2 phenotype polarization. Biochem Biophys Res Commun. (2023) 646:19–29. doi: 10.1016/j.bbrc.2022.12.076, PMID: 36701891

[56] Wang L Zhang Y Yue J Zhou R. The role of ubiquitination on macrophages in cardiovascular diseases and targeted treatment. Int J Mol Sci. (2025) 26:4260. doi: 10.3390/ijms26094260, PMID: 40362498 PMC12072125

[57] Xia X Xu Q Liu M Chen X Liu X He J. Deubiquitination of CD36 by UCHL1 promotes foam cell formation. Cell Death Dis. (2020) 11:636. doi: 10.1038/s41419-020-02888-x, PMID: 32801299 PMC7429868

[58] Liu Y Zhang X Yu L Cao L Zhang J Li Q. E3 ubiquitin ligase RNF128 promotes Lys63-linked polyubiquitination on SRB1 in macrophages and aggravates atherosclerosis. Nat Commun. (2025) 16:2185. doi: 10.1038/s41467-025-57404-6, PMID: 40038329 PMC11880400

[59] Mizuno T Hayashi H Kusuhara H. Cellular cholesterol accumulation facilitates ubiquitination and lysosomal degradation of cell surface–resident ABCA1. Arterioscler Thromb Vasc Biol. (2015) 35:1347–56. doi: 10.1161/ATVBAHA.114.305182, PMID: 25838426

[60] Aleidi SM Yang A Sharpe LJ Rao G Cochran BJ Rye K-A. The E3 ubiquitin ligase, HECTD1, is involved in ABCA1-mediated cholesterol export from macrophages. Biochim Biophys Acta Mol Cell Biol Lipids. (2018) 1863:359–68. doi: 10.1016/j.bbalip.2017.12.011, PMID: 29306077

[61] A-González N Castrillo A. Liver X receptors as regulators of macrophage inflammatory and metabolic pathways. Biochim Biophys Acta. (2011) 1812:982–94. doi: 10.1016/j.bbadis.2010.12.015, PMID: 21193033

[62] Pehkonen P Welter-Stahl L Diwo J Ryynänen J Wienecke-Baldacchino A Heikkinen S. Genome-wide landscape of liver X receptor chromatin binding and gene regulation in human macrophages. BMC Genomics. (2012) 13:50. doi: 10.1186/1471-2164-13-50, PMID: 22292898 PMC3295715

[63] Zelcer N Hong C Boyadjian R Tontonoz P. LXR regulates cholesterol uptake through Idol-dependent ubiquitination of the LDL receptor. Science. (2009) 325:100–4. doi: 10.1126/science.1168974, PMID: 19520913 PMC2777523

[64] Jiang L-Y Jiang W Tian N Xiong Y-N Liu J Wei J. Ring finger protein 145 (RNF145) is a ubiquitin ligase for sterol-induced degradation of HMG-CoA reductase. J Biol Chem. (2018) 293:4047–55. doi: 10.1074/jbc.RA117.001260, PMID: 29374057 PMC5857978

[65] Kim KH Yoon JM Choi AH Kim WS Lee GY Kim JB. Liver X receptor ligands suppress ubiquitination and degradation of LXRalpha by displacing BARD1/BRCA1. Mol Endocrinol. (2009) 23:466–74. doi: 10.1210/me.2008-0295, PMID: 19164445 PMC5419272

[66] Wang Y Li N Zhang X Horng T. Mitochondrial metabolism regulates macrophage biology. J Biol Chem. (2021) 297:100904. doi: 10.1016/j.jbc.2021.100904, PMID: 34157289 PMC8294576

[67] Bhatia D Chung K-P Nakahira K Patino E Rice MC Torres LK. Mitophagy-dependent macrophage reprogramming protects against kidney fibrosis. JCI Insight. (2019) 4:e132826. doi: 10.1172/jci.insight.132826, PMID: 31639106 PMC6962025

[68] Mouton-Liger F Rosazza T Sepulveda-Diaz J Ieang A Hassoun S Claire E. Parkin deficiency modulates NLRP3 inflammasome activation by attenuating an A20-dependent negative feedback loop. Glia. (2018) 66:1736–51. doi: 10.1002/glia.23337, PMID: 29665074 PMC6190839

[69] Itoh K Wakabayashi N Katoh Y Ishii T O’Connor T Yamamoto M. Keap1 regulates both cytoplasmic-nuclear shuttling and degradation of Nrf2 in response to electrophiles. Genes Cells. (2003) 8:379–91. doi: 10.1046/j.1365-2443.2003.00640.x, PMID: 12653965

[70] Potteti HR Venkareddy LK Noone PM Ankireddy A Tamatam CR Mehta D. Nrf2 regulates anti-inflammatory A20 deubiquitinase induction by LPS in macrophages in contextual manner. Antioxidants. (2021) 10:847. doi: 10.3390/antiox10060847, PMID: 34073293 PMC8228212

[71] Romagnoli A Di Rienzo M Petruccioli E Fusco C Palucci I Micale L. The ubiquitin ligase TRIM32 promotes the autophagic response to Mycobacterium tuberculosis infection in macrophages. Cell Death Dis. (2023) 14:505. doi: 10.1038/s41419-023-06026-1, PMID: 37543647 PMC10404268

[72] Lawrence DW Gullickson G Kornbluth J. E3 ubiquitin ligase NKLAM positively regulates macrophage inducible nitric oxide synthase expression. Immunobiology. (2015) 220:83–92. doi: 10.1016/j.imbio.2014.08.016, PMID: 25182373 PMC4278644

[73] Hoffpauir CT Bell SL West KO Jing T Wagner AR Torres-Odio S. TRIM14 Is a Key Regulator of the Type I IFN Response during *Mycobacterium tuberculosis* Infection. J Immunol. (2020) 205:153–67. doi: 10.4049/jimmunol.1901511, PMID: 32404352 PMC7313415

[74] Tang S Lu C Meng Z Ye Z Qin Y Na N. USP22 enhances atherosclerotic plaque stability and macrophage efferocytosis by stabilizing PPARγ. Commun Biol. (2025) 8:678. doi: 10.1038/s42003-025-08116-6, PMID: 40301680 PMC12041205

[75] Wu H Gao W Ma Y Zhong X Qian J Huang D. TRIM25-mediated XRCC1 ubiquitination accelerates atherosclerosis by inducing macrophage M1 polarization and programmed death. Inflammation Res. (2024) 73:1445–58. doi: 10.1007/s00011-024-01906-4, PMID: 38896288

[76] Kobayashi A Kang M-I Okawa H Ohtsuji M Zenke Y Chiba T. Oxidative stress sensor Keap1 functions as an adaptor for Cul3-based E3 ligase to regulate proteasomal degradation of Nrf2. Mol Cell Biol. (2004) 24:7130–9. doi: 10.1128/MCB.24.16.7130-7139.2004, PMID: 15282312 PMC479737

[77] Tao Y Zhao Q Lu C Yong W Xu M Wang Z. Melatonin suppresses atherosclerosis by ferroptosis inhibition via activating NRF2 pathway. FASEB J. (2024) 38:e23678. doi: 10.1096/fj.202400427RR, PMID: 38780199

[78] Wang Y Hollingsworth LR Sangaré LO Paredes-Santos TC Krishnamurthy S Penn BH. Host E3 ubiquitin ligase ITCH mediates Toxoplasma gondii effector GRA35-triggered NLRP1 inflammasome activation and cell-autonomous immunity. mBio. (2024) 15:e03302–23. doi: 10.1128/mbio.03302-23, PMID: 38376248 PMC10936166

[79] Miyauchi S Arimoto K Liu M Zhang Y Zhang D-E. Reprogramming of tumor-associated macrophages via NEDD4-mediated CSF1R degradation by targeting USP18. Cell Rep. (2023) 42:113560. doi: 10.1016/j.celrep.2023.113560, PMID: 38100351 PMC10822669

[80] Feng Y Zou X Huang J Huang Z Kuang G Jiang Y. The E3 ubiquitin ligase MAEA promotes macrophage phagocytosis and inhibits gastrointestinal cancer progression by mediating PARP1 ubiquitination and degradation. Int J Biol Sci. (2025) 21:1784–800. doi: 10.7150/ijbs.102796, PMID: 39990651 PMC11844278

[81] Oishi Y Sohrabi Y Xiao P. Editorial: Metabolic regulation of macrophage functions in inflammation. Front Immunol. (2024) 15:1369896. doi: 10.3389/fimmu.2024.1369896, PMID: 38380325 PMC10877059

[82] Gauthier T Chen W. Modulation of macrophage immunometabolism: A new approach to fight infections. Front Immunol. (2022) 13:780839. doi: 10.3389/fimmu.2022.780839, PMID: 35154105 PMC8825490

[83] Dussold C Zilinger K Turunen J Heimberger AB Miska J. Modulation of macrophage metabolism as an emerging immunotherapy strategy for cancer. J Clin Invest. (2024) 134:e175445. doi: 10.1172/JCI175445, PMID: 38226622 PMC10786697

